# Anatomical factors influencing temporomandibular joint clicking in young adults: temporomandibular joint structure disorder or lateral pterygoid muscle dysfunction?

**DOI:** 10.3389/fbioe.2024.1337267

**Published:** 2024-05-27

**Authors:** Dan Luo, Hua Yang, Mujie Yuan, Dashan Wang, Cheng Qiu, Ruizhi Zhou, Yudong Gao, Ruijie Xu, Jianjun Yang, Zexian Xu

**Affiliations:** ^1^ Department of Oral and Maxillofacial Surgery, The Affiliated Hospital of Qingdao University, Qingdao, Shandong, China; ^2^ School of Stomatology of Qingdao University, Qingdao, Shandong, China; ^3^ Dental Digital Medicine and 3D Printing Engineering Laboratory of Qingdao University, Qingdao, Shandong, China; ^4^ Department of Stomatology, People’s Hospital of Lanling County, Linyi, Shandong, China; ^5^ Department of Oral Implantology, The Affiliated Hospital of Qingdao University, Qingdao, Shandong, China; ^6^ Department of Orthopaedic Surgery, Qilu Hospital, Cheeloo College of Medicine, Shandong University, Qingdao, Shandong, China; ^7^ Department of Radiology, The Affiliated Hospital of Qingdao University, Qingdao, Shandong, China; ^8^ School of Electronic Information, Qingdao University, Qingdao, Shandong, China

**Keywords:** temporomandibular joint clicking, joint structure, masticatory muscle function, lateral pterygoid muscle, texture analysis

## Abstract

**Objective:** This study aimed to investigate the selected anatomical factors that can potentially influence temporomandibular joint (TMJ) clicking in young adults by assessing TMJ structures and lateral pterygoid muscle (LPM) function using magnetic resonance imaging (MRI).

**Methods:** The patients were divided into four groups: the healthy control group; the clicking on mouth opening group; the clicking on mouth closing group; and the clicking on mouth opening and closing group. Additionally, we used clinical palpation to evaluate the masticatory muscles' functional state and employed MRI using the OCOR-T1WI-FSE-CLOSED, OSAG-PDW-FSE-CLOSED, and OSAG-PDW-FSE-OPEN sequences to analyze the texture of the lateral pterygoid muscle (LPM).

**Results:** The proportion of any articular disc or condylar morphology class did not differ significantly between the TMJ clicking and HC groups. The articular disc position did not differ significantly between the TMJ clicking and HC groups. In the TMJ clicking group, the presence of masticatory muscle dysfunction differed significantly between the clicking and non-clicking sides. Moreover, the LPM accounted for the highest proportion among masticatory muscles with tenderness in all TMJ clicking subgroups (77.78%–100%). Therefore, in the TMJ clicking group, the LPM texture was less defined, more uniform in gray scale, and more similar to local texture (*p* < 0.0001).

**Conclusion:** The occurrence of TMJ clicking in young adults is unrelated to the TMJ structure but related to the function of masticatory muscles, particularly the LPM.

## 1 Introduction

Temporomandibular disorders (TMDs) are common among young adults and middle-aged people (Manfredini, Piccotti, Ferronato and Guarda-Nardini, 2010; Gauer and Semidey, 2015; Ujin Yap, Cao, Zhang, Lei and Fu, 2021). The main clinical manifestations are maxillofacial pain, abnormal mandibular movements, and temporomandibular joint (TMJ) clicking (Gauer and Semidey, 2015; F; Liu and Steinkeler, 2013). To date, the etiology of TMDs is unknown (F. Liu and Steinkeler, 2013; Murphy, MacBarb, Wong and Athanasiou, 2013). At present, we know that many etiological factors may lead to the development of TMDs. Some scholars use biopsychosocial models to explain the multifactorial origin of TMDs. The biopsychosocial nature of TMDs has been confirmed by the Orofacial Pain: Prospective Evaluation and Risk Assessment (OPPERA) study and many other studies around the world (Sanders et al., 2013; Slade et al., 2016; Ostrom et al., 2017; Fillingim et al., 2018; Slade et al., 2020; Ao et al., 2024). The OPPERA study focuses on baseline measurements from six risk domains: 1) sociodemographic variables, 2) measures of experimental pain sensitivity, 3) measures of autonomic function, 4) measures of psychological functioning, 5) measures of general health status, and 6) clinical orofacial characteristics (Smith et al., 2011; Bair et al., 2013; Wieckiewicz et al., 2022). In recent years, most young patients with TMDs admitted to our clinic have complained of TMJ clicking, which may occur when opening or closing the mouth (Gazal, 2020). Without timely treatment, TMDs may progress to cause limited mouth opening and TMJ pain, leading to condylar bone destruction and osteoarthrosis, seriously affecting the quality of life of the patients (Poluha, De la Torre Canales, Bonjardim and Conti, 2022).

Anterior disk displacement has been implicated in the etiology of TMJ clicking (Altaweel, Elsayed, Baiomy, Abdelsadek and Hyder, 2019). TMJ clicking is the most common clinical symptom and sign of anterior disc displacement with reduction (ADDwR), with other symptoms including TMJ pain, lock jaw, and abnormal mouth opening (Altaweel et al., 2019; Shen, Chen, Xie, Zhang and Yang, 2019). However, some young adults with TMDs present with an articular disk position in the anterior superior part of the condylar roof, without clinical symptoms of TMDs, such as TMJ clicking (Luo, Yang, et al., 2022a). Nevertheless, most young patients with TMJ clicking are not diagnosed with ADDwR. A clinical examination of these patients reveals that tenderness is more common in the lateral pterygoid muscle (LPM) than in the other masticatory muscles (masseter, temporalis, and medial pterygoid muscles). However, to date, the relationship between masticatory muscle function and TMJ clicking has not been elucidated.

This study aimed to investigate the selected anatomical factors that can potentially influence TMJ clicking in young adults by assessing TMJ structures and LPM function using magnetic resonance imaging (MRI). To explore the etiology of TMJ clicking, we selected young adults with a recent onset of TMJ clicking who had a recent history of a TMD without clinical symptoms other than TMJ clicking (TMJ pain, difficulty in mouth opening, and jaw deviation). We assessed the TMJ structure and masticatory muscle function to analyze the etiology of TMJ clicking in young adults.

## 2 Materials and methods

The study protocol was approved by the Ethics Committee of the Affiliated Hospital of Qingdao University (approval no.: QYFYWZLL26847). All included patients signed an informed consent form after being explained the study protocol.

To ensure the reliability of the results of the study, clinical and MRI examinations of the patients were performed by three stomatologists with more than 15 years of clinical experience in the diagnosis and treatment of TMDs. In addition, MRI was performed for all patients by the same radiologist with 15 years of experience. The order of MRI scans was randomized for the patients at the time of interpretation. In the case of discrepancy, another interpretation was made, and the result with the highest frequency was selected for the final analysis. The intraclass correlation coefficient (ICC) was calculated to analyze the consistency of the results measured by the three stomatologists (Koo and Li, 2016).

### 2.1 Patient selection

**FIGURE 1:**
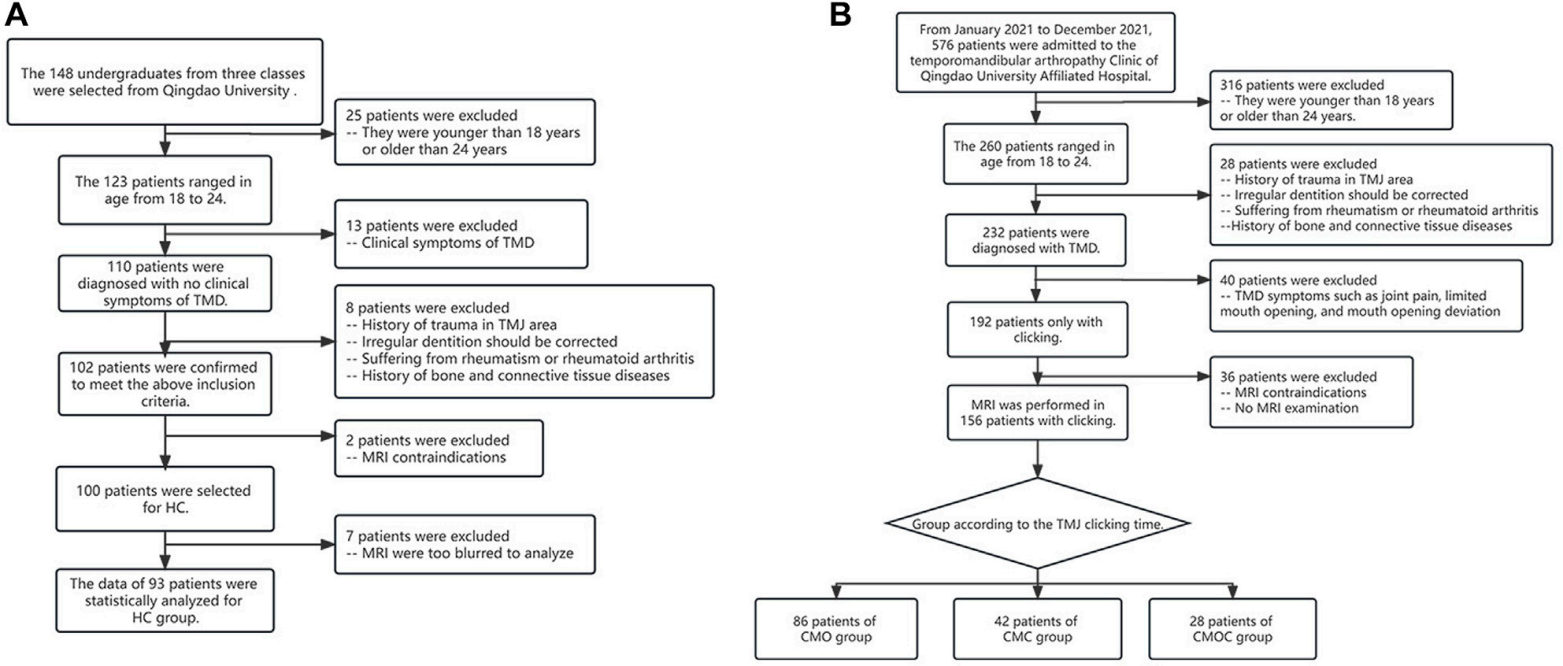
Process of patient selection and classification. **(A)** Allocation of patients in the healthy control (HC) group. **(B)** Allocation of patients in the clicking on mouth opening (CMO), clicking on mouth closing (CMC), and clicking on mouth opening and closing (CMOC) groups.

The patients were divided into four groups (Figure 1): the healthy control (HC) group, the clicking on mouth opening (CMO) group, the clicking on mouth closing (CMC) group, and the clicking on mouth opening and closing (CMOC) group.

We selected 100 freshmen at the Qingdao University for the HC group (Figure 1A). The inclusion criteria were as follows: (1) no history of TMDs or TMJ injury and no symptoms or signs of TMDs, as ascertained by an experienced clinical specialist; (2) age, 18–24 years; (3) gender is not limited, and the women are not pregnant (Minervini et al., 2023b); (4) balanced occlusion, without any history of orthodontic treatment; (5) no history of rheumatism, rheumatoid arthritis, and bone or connective tissue diseases; (6) no history of prolonged jaw widening, such as from dental treatment or singing; (7) no contraindication to MRI, such as metal implants *in vivo* or claustrophobia; (8) no experience of taking pills/drugs that will affect the neuromuscular system and/or skeletal system; (9) no use of stimulants; and (10) no active malignancy, severe mental and neurological disorders, and people addicted to alcohol and/or drugs.

We selected 576 patients with TMDs who had visited the Department of Oral and Maxillofacial Surgery, Qingdao University, from January to December 2021 (Figure 1B). The patients experienced no clinical symptoms of TMDs other than TMJ clicking, such as TMJ pain or discomfort, limited mouth opening, or jaw deviation, indicating a recent onset of TMDs. The remaining inclusion criteria of TMJ clicking patients were the same as those in 2–10 of the HC group. To determine the presence of clicking, the stomatologists placed the fifth finger into the external auditory canal of the participant, with the stomatologist’s ear approximately 5 cm away from the TMJ, and asked the participant to open and close their mouth. Each stomatologist examined each participant thrice, yielding a total of nine examinations per participant. TMJ clicking was determined as positive in the presence of clicking sounds in more than two examinations. Finally, the patients were divided into the CMO, CMC, and CMOC groups.

### 2.2 MRI examination

MRI examination and analysis were performed using the Siemens MAGNETOM 3T Prisma scanner (Siemens, Erlangen, Germany), which uses 60-channel head coils instead of conventional surface coils to yield improved overall spatial information about the masticatory muscle and soft tissue structures. The participants were placed in the supine position, and their heads were scanned with the positioning line aligned with the external auditory canal. Oblique sagittal proton density-weighted fast spin echo images of the TMJ in the closed- and open-mouth positions (OSAG-PDW-FSE-CLOSED and OSAG-PDW-FSE-OPEN, respectively) and oblique coronal T1-weighted images in the closed-mouth position (OCOR-T1WI-FSE-CLOSED) were obtained.

The OSAG-PDW-FSE-CLOSED and OSAG-PDW-FSE-OPEN sequences were applied in the parallel acquisition technology with the following parameters: excitation time, 3 s; repetition time, 2,070 ms; echo time, 28 ms; field of view, 120 mm × 120 mm; matrix, 192 × 144; and planar resolution, 0.6 mm × 0.6 mm. Alternatively, the OCOR-T1WI-FSE sequence was applied in the parallel acquisition technology with the following parameters: number of excitations, three; repetition time, 550 ms; echo time, 6.8 ms; field of view, 120 × 120 mm; matrix, 192 × 144; and planar resolution, 0.6 mm × 0.6 mm. A total of 16 images were captured using each scanning sequence. Each image layer had a thickness of 2 mm, and the interval between the layers was 10%. Eight images each for the right and left TMJs were acquired, with each sequence consisting of 16 images. The slice thickness of each image was 2 mm, and the gap was 10%.

### 2.3 TMJ structure on MRI scans

**FIGURE 2:**
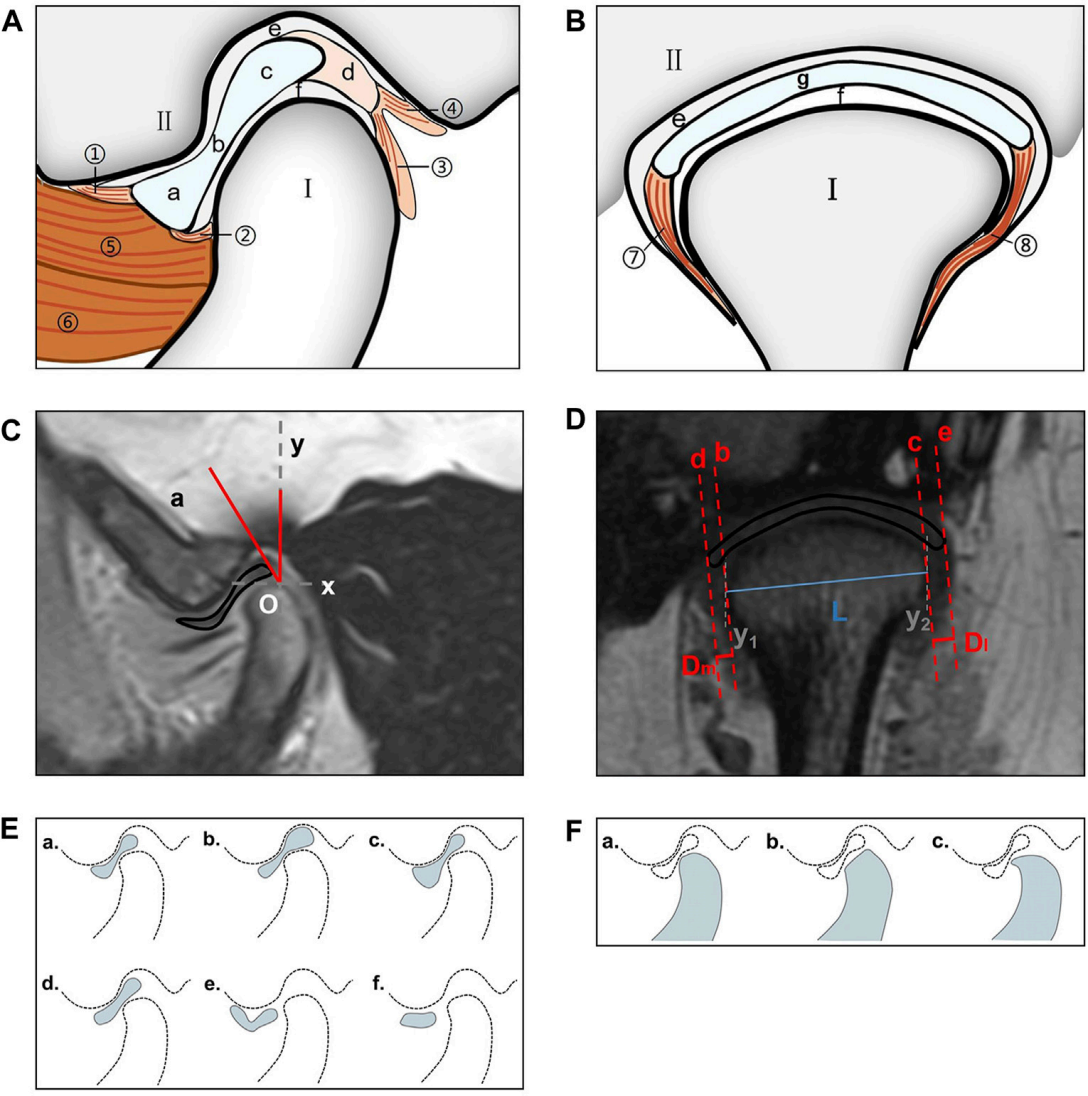
TMJ structure and measurement method. **(A)** View from the oblique sagittal plane; I: condylar process; II: glenoid fossa; a: anterior band; b: intermediate zone; c: posterior band; d: bilaminar zone; e: superior joint cavity; f: inferior joint cavity; ①: anterior temporal attachment; ②: anterior mandibular attachment; ③: posterior mandibular attachment; ④: posterior temporal attachment; ⑤: superior head of the LPM; ⑥: inferior head of the LPM. **(B)** View from the oblique coronal plane. I: condylar process; II: glenoid fossa; e: superior joint cavity; f: inferior joint cavity; g: articular disc; ⑦: medial disc ligament; ⑧: lateral disc ligament. **(C)** Use of the condylar apex method to measure the articular disc position (∠aOy). O: condylar apex. **(D)** The condylar length “L” (“y1–y2” is the vertical line passing through both sides of the condyle; “L” is the line connecting the intersection point of the two perpendicular lines and the condyle), medial displacement distance “Dm,” and lateral displacement distance “Dl” of the articular disk were measured in the oblique coronal plane (“b–c” is the tangent drawn at the intersection of both sides of the condyle; “d–e” is parallel to “c–d” and passes through both ends of the disk; and “Dm–Dl” is the distance between “e–c” and “d–f”). **(E)** Articular disc morphology; a: biconcave; b: thickening posterior; c: thickening anterior; d: biplanar; e: biconvex; f: folded. **(F)** Condylar morphology; a: round; b: flattened; c: beak-like. TMJ, temporomandibular joint; LPM, lateral pterygoid muscle.

Figures 2A,B show the TMJ structure. MRI scans with a motion artifact were excluded.

The articular disk–condylar position relationship was determined (Luo, Yang, et al., 2022b). Using AutoCAD 2005 (accuracy, 0.01; Autodesk, San Rafael, CA, United States), the articular disk–condyle angle in the oblique sagittal plane was measured with the condylar apex method (Figure 2C). Centricity DICOM Viewer (precision, 0.01 mm; GE HealthCare, Chicago, United States) software was used to measure the medial and lateral displacements of the oblique coronal articular disk relative to the condyle (Figure 2D) (Eberhard, Giannakopoulos, Rohde and Schmitter, 2013).

The articular disk and condylar morphologies were determined in the oblique sagittal plane (Luo, Qiu, et al., 2022a). The articular disk morphology was divided into six categories: biconcave, thickening posterior, thickening anterior, biplanar, biconvex, and folded (Figure 2E). The condylar morphology was divided into three categories: round, flattened, and beak-like (Figure 2F).

### 2.4 Masticatory muscle function on MRI

**FIGURE 3:**
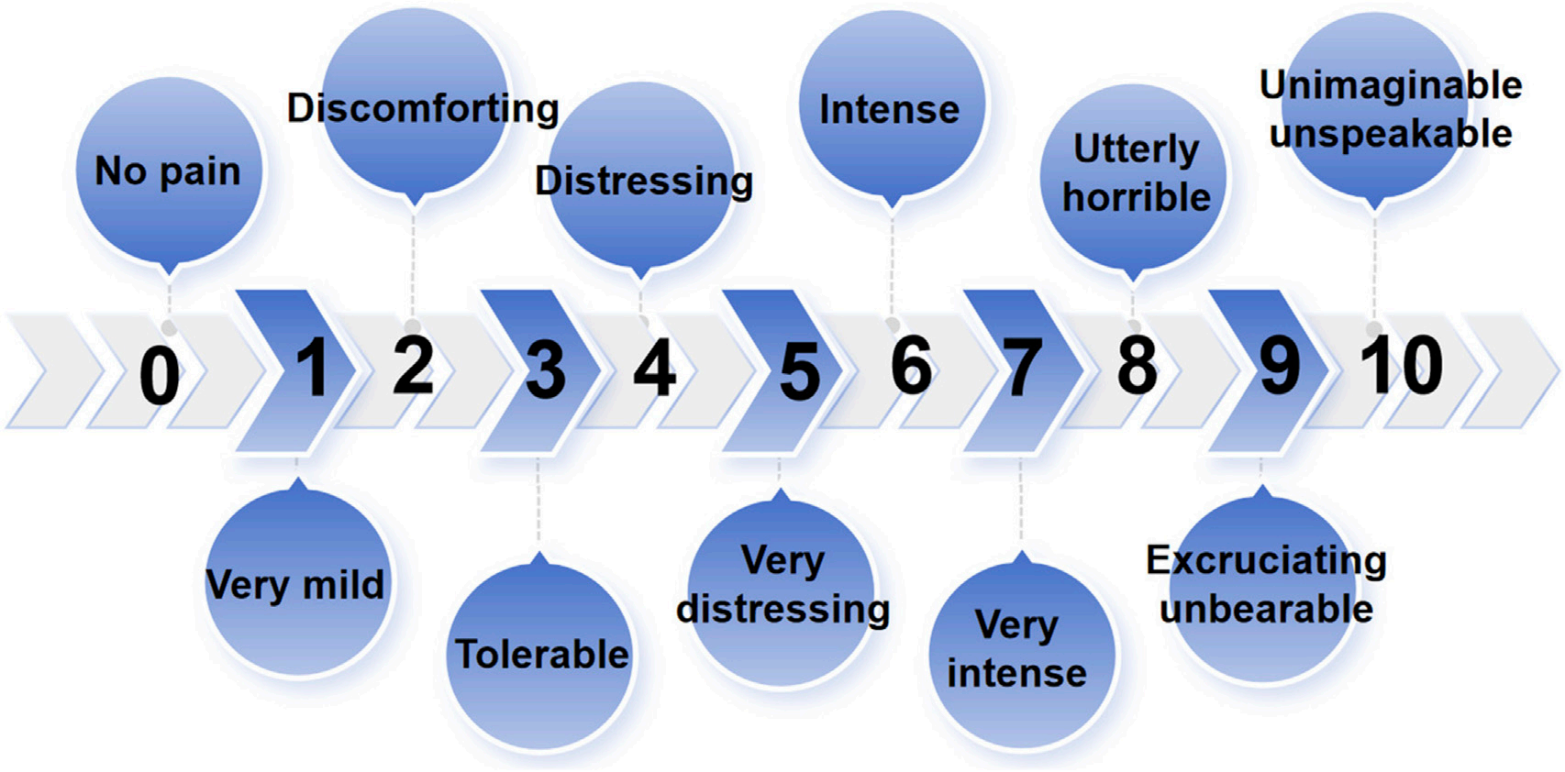
Numerical rating scale (NRS) is used to quantify the tenderness felt by the patients.

Tenderness of the TMJ area and masticatory muscle: The sites examined included the temporomandibular joint area, the masseter muscle (MM), the temporalis muscle (TM), the medial pterygoid muscle (MPM), and the lateral pterygoid muscle (LPM). During the tenderness examination, the touch pressure diagnosis force was approximately 1 kg, and the duration was approximately 3 s. A numerical rating scale (NRS) was used to quantify the tenderness felt of the patients (Figure 3). The patients indicated the number corresponding to the pain intensity, and each stomatologist recorded the examination results and took the average of the three examination results as the basis for follow-up statistical analysis.

**FIGURE 4:**
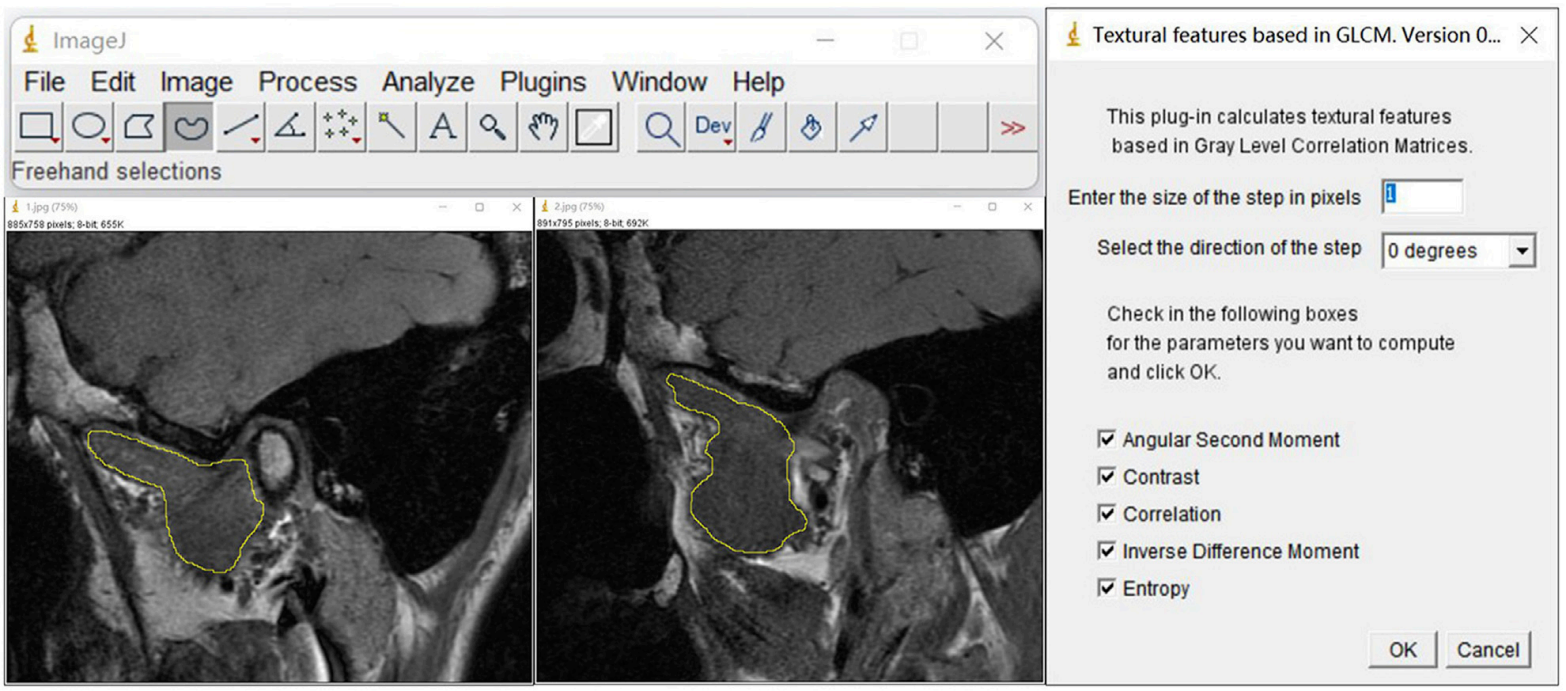
TA parameters of the LPM obtained with the OSAG-PDW-FSE-CLOSED and OSAGPDW-FSE-OPEN MRI sequences. The first step was the selection of the region of interest of the LPM with the graph selection tool, avoiding the inclusion of peripheral blood vessels, nerves, and other structures. The LPM boundary is outlined with yellow in the three images. The second step was the selection of “plugins” for the TA. The selected conditions are shown. The corresponding results were calculated by checking the five TA parameters (angular second moment, contrast, correlation, inverse difference moment, and entropy). LPM, lateral pterygoid muscle; TA, texture analysis.

Furthermore, we used open-source ImageJ (1.51j8) (https://imagej.net/ij/index.html) software with the gray-level co-occurrence matrix plug-in version 0.4 to perform a texture analysis (TA) of the LPM using MRI with the OSAG-PDW-FSE-CLOSED and OSAG-PDW-FSE-OPEN sequences. This plug-in calculates textural features based in gray-level correlation matrices. The size of the step entered in pixels is 1, the selected direction of the step is 0 degrees, and the five TA parameters are selected: angular second moment (ASM), contrast, correlation, inverse difference moment (IDM), and entropy. The three stomatologists outlined the regions of interest with the freehand selection method, including as much of the LPM as possible and avoiding the surrounding structures. Then, they collected data on the five TA parameters (Figure 4) (Luo et al., 2023a). The radiologists obtained one measurement each, and the average value of the measurements was used for statistical analyses. TA can reveal subtle changes in tissues and muscles that cannot be visually observed and convert them into corresponding quantifiable parameters. Comparing these parameters among the groups could accurately reveal the characteristics of the LPM in each group. We measured five TA parameters: ASM, contrast, correlation, IDM, and entropy. These parameters represent different local gray levels in the image (Table 1).

**TABLE 1:** Meaning of five parameters of TA.

Parameter	Local conditions in the image
ASM	Uniformity of gray distribution and the texture of an image, with higher values indicating more uniform gray levels
Contrast	Local changes in an image, which can reflect the sharpness and depth of texture grooves. A higher value indicates a clearer texture
Correlation	Similarity in the degree of the gray level in a row or a column of the measurement image. A higher value indicates a stronger correlation of the local gray level
IDM	Clarity and regularity of the texture. A larger value indicates a clearer and more regular texture of the image
Entropy	Randomness of the information contained in an image, with a larger value indicating more complex gray distribution of the image

TA, texture analysis; ASM, angular second moment; IDM, inverse differential moment.

### 2.5 Consistency analysis of the results

Since the TA of the LPM is more complex than that of the other masticatory muscles, the results obtained by the three stomatologists were expected to show the greatest difference for the LPM. Because the results of the five TA parameters were obtained simultaneously in a single measurement, we could choose any parameter to calculate the ICC. The ICC of the ASM, among all TA parameters, in the open- and closed-mouth positions of patients in the HC group represented the consistency of results measured by the three stomatologists.

### 2.6 Statistical analysis

We performed all the statistical analyses using SPSS version 20.0 (IBM SPSS, Inc., Armonk, NY, United States) and GraphPad Prism 8.0.1 (Dotmatics, Boston, MA, United States). Data are expressed as maximum, minimum, mean, and standard deviation. Normally distributed data were compared using one-way analysis of variance (ANOVA) and *t*-test, and non-normally distributed data were compared using the Wilcoxon rank-sum and nonparametric tests. Categorical data were compared using the chi-squared test. Thus, the Wilcoxon rank-sum test was used for the TA parameters ASM, contrast, and correlation. Finally, we analyzed the IDM and entropy using one-way ANOVA and performed an overall statistical analysis using two-way ANOVA.

## 3 Results

### 3.1 Demographic data

All the volunteers in the HC group underwent an MRI examination, seven of whom were excluded because of motion artifact. Patients with TMDs who had imaging errors actively cooperated for repeated MRI examinations until the clinical diagnosis could be obtained based on the MRI findings. Therefore, none of the patients with TMDs were excluded because of the motion artifact. Finally, a total of 249 patients were enrolled, i.e., 93 in the HC group, 86 in the CMO group, 42 in the CMC group, and 28 in the CMOC group (Figure 1). The age or sex ratio did not differ significantly among the groups (Table 2).

**TABLE 2:** Demographics of study groups.

	HC	CMO	CMC	CMOC	*p*-value
	*n* = 93	*n* = 86	*n* = 42	*n* = 28	
Age **(yr, mean ± SD)**	19.87 ± 1.45	20.47 ± 1.83	20.48 ± 1.63	20.32 ± 1.85	0.073
Sex (n, %)					
Female	57 (61.29%)	54 (62.80%)	27 (64.29%)	17 (60.71%)	0.985
Male	36 (38.71%)	32 (37.20%)	15 (35.71%)	11 (39.29%)	
Side (n, %)					
Clicking	0	102 (59.30%)	57 (67.86%)	39 (69.64%)	
Non-clicking	186 (100%)	70 (40.70%)	27 (32.14%)	17 (30.36%)	

HC, healthy control; CMO, clicking on mouth opening; CMC, clicking on mouth closing; CMOC, clicking on mouth opening and closing.

### 3.2 TMJ structure

Table 3 shows the articular disk position in the oblique sagittal plane of the patients in each group in the closed-mouth position. The articular disk position differed significantly between men and women in the HC group (*p* = 0.0463) but not in the other three groups. In addition, it did not differ significantly between the clicking and non-clicking sides of the patients in the CMO, CMC, or CMOC group (Table 3). Finally, it did not differ significantly in the oblique coronal plane between the clicking and HC groups (*p* > 0.05; Table 4).

**TABLE 3:** Oblique sagittal disk position as measured by the condylar apex methods.

	HC				CMO				CMC				CMOC				*p*-value

	Minimum	Maximum	Mean	SD	Minimum	Maximum	Mean	SD	Minimum	Maximum	Mean	SD	Minimum	Maximum	Mean	SD	
Total	−25.86	122.67	34.73	18.54	10.06	89.31	32.35	12.47	14.99	62.26	35.65	9.02	14.53	58.57	35.72	9.16	0.2056
**Side**																	
Clicking					10.06	89.31	32.44	13.48	14.99	62.26	35.54	9.19	20.40	58.57	36.17	9.09	0.1235
Non-clicking					14.55	63.47	32.24	10.92	19.36	56.75	35.88	8.81	14.53	51.76	34.68	9.53	0.2583
***p*-value (**clicking vs. non-clicking)					0.9184				0.8703				0.5798				

HC, healthy control; CMO, clicking on mouth opening; CMC, clicking on mouth closing; CMOC, clicking on mouth opening and closing.

**TABLE 4:** Oblique coronal disk position as measured.

		HC				CMO				CMC						CMOC				*p*-value
		Minimum	Maximum	Mean	SD	Minimum	Maximum	Mean	SD	Minimum	Maximum		Mean		SD	Minimum	Maximum	Mean	SD	
Medial distance (Dm)	Total	−1.467	4.427	0.661	1.194	−1.558	3.253	0.652	1.430	−1.553	3.121		0.765		1.377	−1.558	3.043	0.787	1.349	0.8488
	**Side**																			
	Clicking					−1.558	3.253	0.749	1.356	−1.553	3.042		0.779		1.338	−1.558	3.043	0.667	1.357	0.9205
	Non-clicking					−1.523	3.225	0.514	1.526	−1.546	3.121		0.737		1.483	−1.434	2.785	1.062	1.329	0.3733
	***p*-value (**clicking vs. non-clicking)					0.2867				0.8964						0.3182				
						
Lateral distance (Dl)	Total	−2.153	3.390	0.948	1.169	−2.552	4.213	0.857	1.943	−2.435	3.867	1.044		1.664		−2.437	3.865	1.165	1.629	0.5994
	**Side**																			
	Clicking					−2.502	4.213	0.945	1.815	−2.435	3.867	1.184		1.620		−2.437	3.865	1.222	1.700	0.5866
	non-clicking					−2.552	4.182	0.681	2.093	−2.131	3.842	0.749		1.747		−1.626	3.442	1.036	1.493	0.7952
	***p*-value (**clicking vs. non-clicking)					0.3802				0.2661						0.6987				

HC, healthy control; CMO, clicking on mouth opening; CMC, clicking on mouth closing; CMOC, clicking on mouth opening and closing.

**FIGURE 5:**
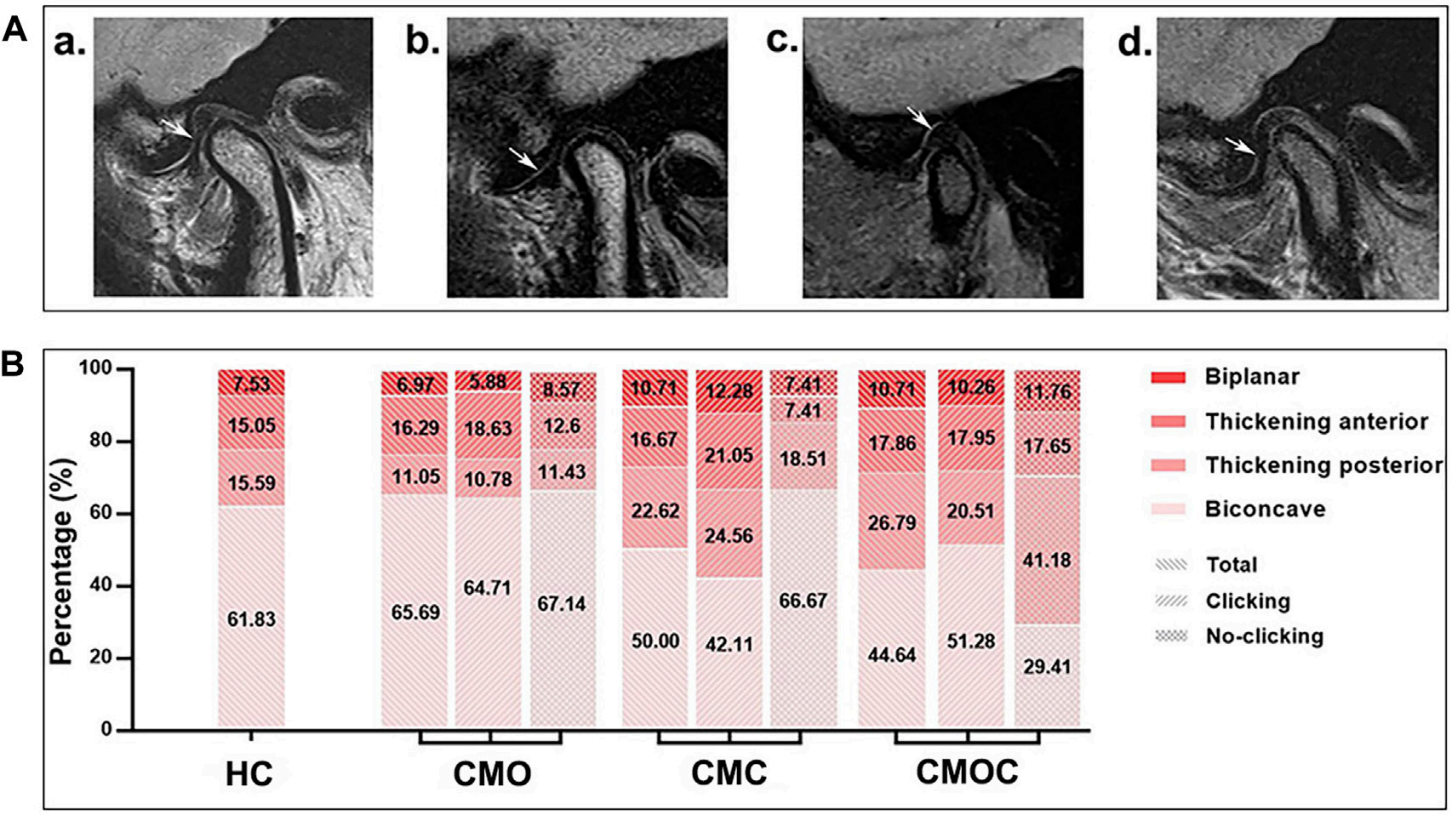
Schematic diagram of the articular disk morphology with distribution rates. **(A) (a)** biconcave; **(b)** thickening posterior; **(c)** thickening anterior; and **(d)** biplanar. **(B)** Proportion of each articular disk morphology class.

**FIGURE 6:**
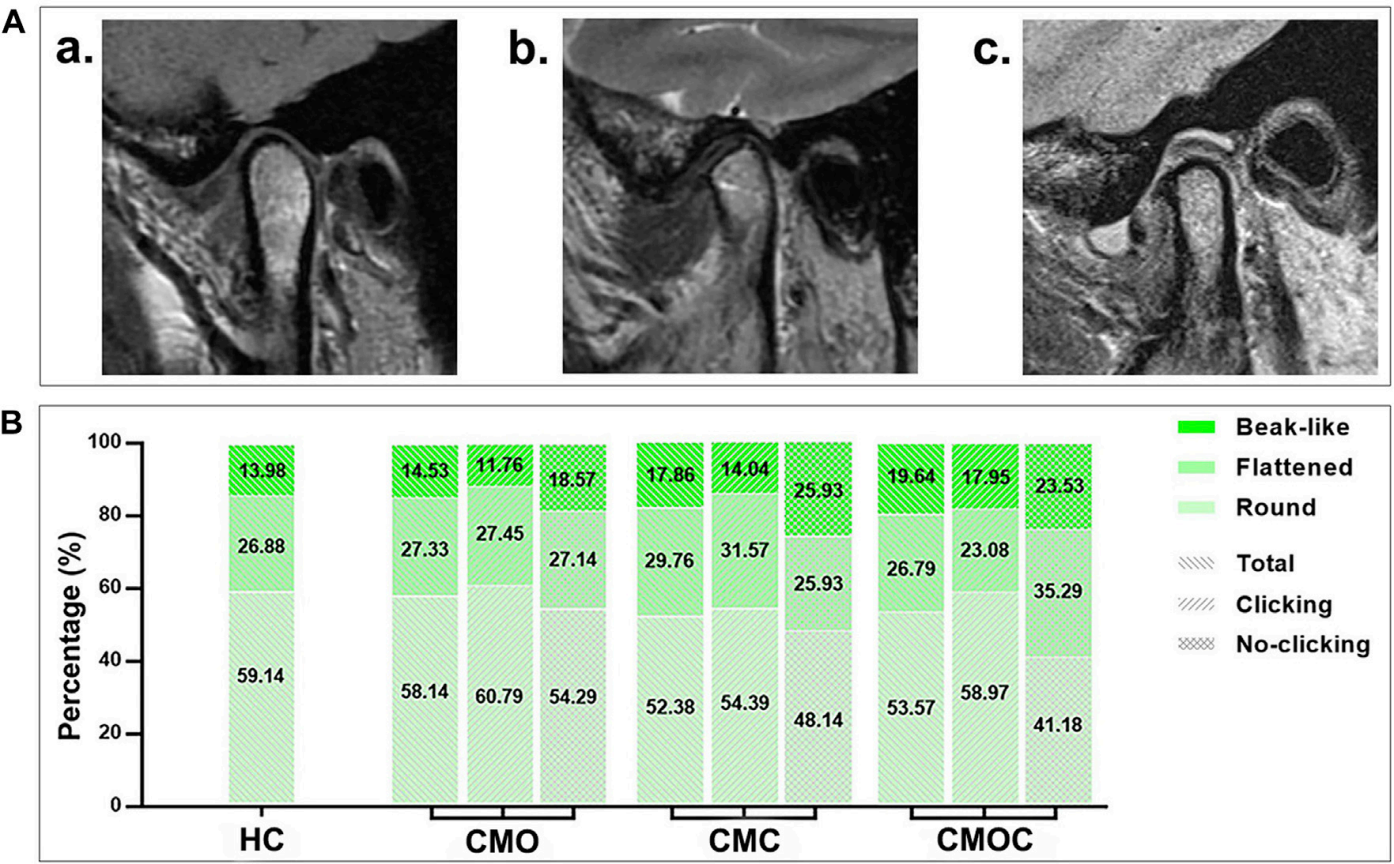
Schematic diagram of the condylar morphology with distribution rates. **(A) (a)** round; **(b)** flattened; **(c)** beak-like. **(B)** Proportion of each condylar morphology class.

The proportion of each articular disk morphology class was statistically similar among the four groups (Figure 5). Biconcave accounted for the largest proportion, followed by thickening posterior, thickening anterior, and biplanar. Likewise, the proportion of each condylar morphology class was statistically similar among the four groups (Figure 6). Round accounted for the largest proportion, followed by flattened and beak-like. The proportion of the previous articular disk (*p* = 0.0883) or condylar (*p* = 0.9086) morphology class did not differ significantly among the four groups. The proportion of the articular disk (*p* = 0.3150) or condylar (*p* = 0.9445) morphology class did not differ significantly among the CMO, CMC, and CMOC groups. In addition, the proportion of the articular disk or condylar morphology class did not differ in the CMO, CMC, and CMOC groups between the clicking and non-clicking sides (*p* > 0.1748).

### 3.3 Masticatory muscle function

Table 5 shows the results of TMJ and masticatory muscle tenderness of the patients in each group. No TMJ area tenderness was observed in the HC group, and there was a significant difference in the NRS value of TMJ area tenderness in the clicking side and the non-clicking side of the CMO group and the CMC group (*p* < 0.0001). In addition, the mean NRS value of LPM tenderness in the HC group was 0.492, and the NRS value of LPM tenderness in the clicking side of the CMO group, CMC group, and CMOC group was about twice that of the NRS value on the non-clicking side, with statistically significant differences (*p* < 0.0001).

**TABLE 5:** Tenderness of temporomandibular joint and masticatory muscle of study groups.

Tenderness	HC	CMO		CMC		CMOC	
	Total (n = 186) (n, %)	Clicking (n = 102) (n, %)	Non-clicking (n = 70) (n, %)	Clicking (n = 57) (n, %)	Non-clicking (n = 27) (n, %)	Clicking (n = 39) (n, %)	Non-clicking (n = 17) (n, %)
TMJ	0	59 (57.84%)	18 (25.71%)	29 (50.88%)	12 (44.44%)	18 (46.15%)	8 (47.06%)
Masseter muscle (MM)	0	62 (60.78%)	22 (31.43%)	36 (63.16%)	15 (55.56%)	19 (48.72%)	12 (70.59%)
Temporalis muscle (TM)	0	66 (64.71%)	25 (35.71%)	31 (54.39%)	12 (44.44%)	22 (56.41%)	9 (52.94%)
Medial pterygoid muscle (MPM)	0	64 (62.75%)	19 (27.14%)	38 (66.67%)	13 (48.15%)	26 (66.67%)	11 (64.71%)
Lateral pterygoid muscle (LPM)	12 (10.0%)	100 (98.04%)	65 (92.86%)	53 (92.98%)	21 (77.78%)	39 (100%)	17 (100%)
***p*-value**		COP		CCP		COCP	
		Clicking	Non-clicking	Clicking	Non-clicking	Clicking	Non-clicking
		<0.0001	<0.0001	<0.0001	0.0740	<0.0001	0.0108

HC, healthy control; CMO, clicking on mouth opening; CMC, clicking on mouth closing; CMOC, clicking on mouth opening and closing.

Table 6 and Table 7 show the results of the TA of the LPM obtained with the OSAG-PDW-FSE-CLOSED and OSAG-PDW-FSE-OPEN MRI sequences, respectively. The results of the TA parameters of the LPM obtained with each sequence differed significantly among all groups (*p* < 0.0001) and between the HC and clicking groups (*p* < 0.0001). On the clicking side in the open-mouth position, only entropy differed significantly among the CMO, CMC, and CMOC groups (*p* = 0.0496). On the clicking side in the closed-mouth position, the correlation, IDM, and entropy differed significantly among the CMO, CMC, and CMOC groups (*p* < 0.0045). In the closed-mouth position, the ASM differed significantly between the clicking and non-clicking sides of the patients in the CMO group (*p* = 0.0101). In the open-mouth position, the correlation differed significantly between the clicking and non-clicking sides of the patients in the CMO group (*p* < 0.0001). Combined with the results of the LPM TA of the above three MRI sequences, according to the meaning analysis of the parameters of TA, the local gray-level difference in the LPM MRI in young TMD patients with clicking symptoms is small, the correlation is strong, and the texture images are fuzzy and irregular. On the other hand, the texture of LPM on the clicking side of the patients was more uniform than that on the non-clicking side, and the correlation of the local gray scale was stronger.

**TABLE 6:** Texture analysis results of lateral pterygoid muscle in OSAG-PDW-FSE-CLOSE magnetic resonance imaging sequence on clicking and non-clicking sides.

		OSAG-PDW-FSE-CLOSE				
		ASM (×10–3)	Contrast	Correlation (×10–3)	IDM	Entropy
HC (*n* = 93 × 2)		0.828 ± 1.092	41.932 ± 8.569	1.276 ± 0.647	0.430 ± 0.158	7.000 ± 0.518
CMO	Clicking (*n* = 102)	1.279 ± 0.393	33.732 ± 8.59	1.601 ± 0.239	0.261 ± 0.022	7.691 ± 0.272
	Non-clicking (*n* = 70)	0.940 ± 0.245	36.028 ± 8.303	1.386 ± 0.233	0.259 ± 0.022	7.708 ± 0.272
CMC	Clicking (*n* = 57)	1.254 ± 0.533	32.248 ± 9.009	1.645 ± 0.160	0.257 ± 0.031	7.749 ± 0.204
	Non-clicking (*n* = 27)	1.086 ± 0.535	30.880 ± 7.094	1.456 ± 0.158	0.260 ± 0.028	7.721 ± 0.171
CMOC	Clicking (*n* = 39)	1.219 ± 0.294	32.534 ± 6.794	1.557 ± 0.108	0.267 ± 0.024	7.796 ± 0.170
	Non-clicking (*n* = 17)	0.934 ± 0.068	34.669 ± 6.855	1.366 ± 0.107	0.267 ± 0.021	7.825 ± 0.148
*p-value*						
Two-way ANOVA (Total)		<0.0001				
		Wilcoxon rank-sum test	Wilcoxon rank-sum test	Wilcoxon rank-sum test	One-way ANOVA	One-way ANOVA
(HC and clicking of CMO, CMC, and CMOC)		<0.0001	<0.0001	<0.0001	<0.0001	<0.0001
(Clicking of CMO, CMC, and CMOC)		0.6144	0.5303	0.1012	0.1323	0.0496
		Nonparametric test	Nonparametric test	Nonparametric test	*t*-test	*t*-test
(Clicking vs. non-clicking of CMO)		0.0101	0.0928	0.7000	0.6665	0.6966
(Clicking vs. non-clicking of CMC)		0.7999	0.4903	0.7607	0.6245	0.5387
(Clicking vs. non-clicking of CMOC)		0.2481	0.2858	0.7729	0.9488	0.5384

HC, healthy control; CMO, clicking on mouth opening; CMC, clicking on mouth closing; CMOC, clicking on mouth opening and closing; OSAG-PDW-FSE-CLOSE, oblique sagittal proton density-weighted fast spin-echo close.

**TABLE 7:** Texture analysis results of lateral pterygoid muscle in OSAG-PDW-FSE-OPEN magnetic resonance imaging sequences on clicking and non-clicking sides.

		OSAG-PDW-FSE-OPEN				
		ASM (×10–3)	Contrast	Correlation (×10–3)	IDM	Entropy
HC (*n* = 93 × 2)		0.457 ± 0.712	32.028 ± 3.596	0.491 ± 0.828	0.391 ± 0.051	6.823 ± 0.394
CMO	Clicking (*n* = 102)	0.912 ± 0.168	27.304 ± 6.234	1.877 ± 0.519	0.263 ± 0.032	7.582 ± 0.288
	Non-clicking (*n* = 70)	0.871 ± 0.214	29.276 ± 7.687	0.846 ± 0.509	0.261 ± 0.032	7.608 ± 0.299
CMC	Clicking (*n* = 57)	0.976 ± 0.369	28.016 ± 5.583	0.755 ± 0.165	0.261 ± 0.021	7.709 ± 0.129
	Non-clicking (*n* = 27)	0.993 ± 0.375	27.083 ± 5.286	0.776 ± 0.142	0.260 ± 0.021	7.724 ± 0.115
CMOC	Clicking (*n* = 39)	0.957 ± 0.086	25.132 ± 6.813	0.749 ± 0.181	0.284 ± 0.026	7.590 ± 0.211
	Non-clicking (*n* = 17)	0.976 ± 0.053	24.663 ± 7.632	0.793 ± 0.188	0.289 ± 0.020	7.552 ± 0.200
*p-value*						
Two-way ANOVA (Total)		<0.0001				
		Wilcoxon rank-sum test	Wilcoxon rank-sum test	Wilcoxon rank-sum test	One-way ANOVA	One-way ANOVA
(HC and clicking of CMO, CMC, and CMOC)		<0.0001	<0.0001	<0.0001	<0.0001	<0.0001
(Clicking of CMO, CMC, and CMOC)		0.2358	0.0723	<0.0001	0.0001	0.0045
		Nonparametric test	Nonparametric test	Nonparametric test	*t*-test	*t*-test
(Clicking vs. non-clicking of CMO)		0.1645	0.0674	<0.0001	0.6146	0.5774
(Clicking vs. non-clicking of CMC)		0.8474	0.4692	0.5659	0.8441	0.6003
(Clicking vs. non-clicking of CMOC)		0.3936	0.8201	0.4164	0.4578	0.5322

HC, healthy control; CMO, clicking on mouth opening; CMC, clicking on mouth closing; CMOC, clicking on mouth opening and closing; OSAG-PDW-FSE-OPEN, oblique sagittal proton density-weighted fast spin-echo open.

### 3.4 Consistency of the results

Table 8 lists the ICCs of ASM of the LPM obtained with the OSAG-PDW-FSE-CLOSED and OSAG-PDW-FSE-OPEN sequences. The ICCs of OSAG-PDW-FSE-CLOSED and OSAG-PDW-FSE-OPEN sequences indicated good reliability.

**TABLE 8:** ICC calculation for ASM parameters of the HC group in the LPM TA of the two MRI sequences.

Variable	Significance	OSAG-PDW-FSE-CLOSE	OSAG-PDW-FSE-OPEN
Sp^2^	Estimate of the variance component due to patients	4.768	3.978
Sj^2^	Estimate of the variance component due to the joint within patients	1.857	2.824
Ss^2^	Estimate of the variance component due to slice within the joint within patients	4.657	4.766
So^2^	Estimate of the variance component due to observers	1.379	1.552
Se^2^	Estimate of the variance component due to sampling errors	1.658	0.852
ICC	Intraclass correlation coefficient	0.788	0.828

ASM, angular second moment; HC, healthy control; LPM, lateral pterygoid muscle; TA, texture analysis; MRI, magnetic resonance imaging; OSAG-PDW-FSE-CLOSE, oblique sagittal proton density-weighted fast spin-echo close; OSAG-PDW-FSE-OPEN, oblique sagittal proton density-weighted fast spin-echo open.

## 4 Discussion

In this study, young adults aged 18–24 years were selected, and those in the CMO, CMC, and CMOC groups experienced only TMJ clicking, indicating the early stage of TMD. According to the current status of global research on the etiology of TMJ clicking, we assessed the TMJ structure and masticatory muscle function in this study (Reid and Greene, 2013; Liu, Lei, Han, Yap and Fu, 2017; Almashraqi, Ahmed, Mohamed and Halboub, 2018; Ohrbach and Michelotti, 2018).

Regarding the structural aspects of the TMJ, we investigated the morphological classes of the articular disk and condyle and the articular disk position. Changes in articular disk morphology occur in internal TMDs and cause TMJ dysfunction. Severe articular disk deformity is significantly associated with TMDs (Taşkaya-Yilmaz and Oğütcen-Toller, 2001; Okur, Ozkiris, Kapusuz, Karaçavus and Saydam, 2012). Hu et al. (Taşkaya-Yilmaz and Oğütcen-Toller, 2001; Hu, Yang and Xie, 2016), in a retrospective study, revealed that morphological changes in the articular disks in the ADDwR group were mainly posterior band enlargement or mild wrinkling, whereas those in the articular disks in the anterior disk displacement without reduction group showed deformity after the follow-up, such as severe wrinkling and shortening, with the double-concave and V-folded morphologies being more likely to shorten the articular disk and displace the anterior part. Among the six articular disk shapes (biconcave, thickening posterior, thickening anterior, biplanar, biconvex, and folded), four (biconcave, thickening posterior, thickening anterior, and biplanar) were found in asymptomatic patients (Luo, Qiu, et al., 2022b). In the present study, only these four articular disk shapes were found, with the proportions not differing significantly between the CMO, CMC, and CMOC and HC groups. In a previous study, the condylar morphology was divided into round, flattened, and beak-like shapes, with most asymptomatic volunteers showing a round condyle (Luo, Qiu, et al., 2022a). Since the condylar shape is closely related to condylar function, the condylar morphology in TMDs differs among patients (Katsavrias and Halazonetis, 2005). For example, flattened and inclined condyles are associated with a strong bite force and masticatory muscle pull (Koyama, Nishiyama and Hayashi, 2007; Alexiou, Stamatakis and Tsiklakis, 2009). In this study, although the proportion of any condylar morphology class did not differ significantly between the clicking (CMO, CMC, and CMOC groups) and HC groups, the proportions of flattened and beak-like morphology were relatively high. This may imply that TMJ clicking is a risk factor for osteoarthritis. The condylar apex method developed in a previous study was more accurate than the condylar midpoint method for articular disk position measurement (Luo, Yang, et al., 2022b). The present study showed no significant difference in the articular disk position between the patients with early-stage TMDs with only clicking symptoms and the asymptomatic volunteers. In addition, we used the method introduced by Eberhard et al. (2013) to measure the articular disk position in the oblique coronal plane and found no significant difference between patients with TMDs with only TMJ clicking and the asymptomatic volunteers. In general, the proportion of any articular disk or condylar morphology class or articular disk position did not differ significantly between the young adults with TMJ clicking and asymptomatic adults. Thus, the present study suggests that the articular disk or condylar morphology or the positional relationship between the articular disk and the condyle, which is closely related to the TMJ structure, does not change significantly in the early stage of the occurrence or development of TMDs.

In this study, we also preliminarily studied masticatory muscle function in patients with TMJ clicking based on the clinical finding of tenderness and MRI findings of the TA parameters of the LPM. The patients in the CMO, CMC, and CMOC groups showed partial positive palpation in the four groups of masticatory muscles. The LPM accounted for the highest proportion of masticatory muscles with tenderness. In particular, in the HC group, 10% of the patients experienced LPM tenderness. However, these patients may have developed TMDs without the onset of the symptoms of TMDs. Due to the anatomical relationship between the LPM and TMJ, the occurrence of TMJ clicking as the initial symptom of TMDs may be closely related to the LPM function. Therefore, in future studies, we aim to investigate the LPM function as an etiologic factor for the initial onset of TMDs. From the aforementioned examination of the masticatory muscles, accurate information about LPM dysfunction could not be obtained.

In recent years, with the rapid development of soft-tissue MRI-related research, TA parameters on MRI have increasingly been accepted (Herlidou, Rolland, Bansard, Le Rumeur and de Certaines, 1999; Harrison et al., 2010). In this study, we performed the TA of the LPM using MRI to avoid the visual differences among the researchers, obtain quantifiable parameters, and explore the functional information about the LPM. We found that the LPM texture in the closed- and open-mouth positions of the young patients with TMJ clicking differed significantly from that of the asymptomatic young adults. Combining the five TA parameters revealed that patients with TMJ clicking showed reduced texture complexity and regularity and more similar local textures in the LPM than asymptomatic young adults. Comparing the LPM texture between the clicking and non-clicking sides in each clicking group revealed that the LPM texture on the clicking side was less clear, more uniform in the gray scale, and more similar in local texture. The results of the TA of LPM shown by MRI may correspond to the function and inflammatory status of LPM. In the normal state of function, the texture of muscle tissue is clear, and the gray contrast in local areas is more obvious. However, in the inflammatory state, muscle tissue is infiltrated by inflammatory cells, along with local muscle tissue edema, which can reduce the texture complexity of the muscle tissue and render the texture unclear and the local gray scale more similar (Herlidou et al., 1999). In a previous study, the TA parameters of the LPM were correlated with the articular disk displacement in patients with TMD (Luo et al., 2023b). When patients with TMD experienced TMJ clicking, the LPM texture was blurred, and the local texture was more similar, which may be caused by the inflammation of the LPM. Since clicking is the most common initial symptom in TMD patients, we can analyze the inflammatory state in the initial stage of TMD patients through the texture characteristics of LPM combined with the results of this study. In addition, the prognosis of TMD patients after treatment can also be further studied by the TA of LPM. In the future, our research group will further elaborate the relationship between the texture changes in LPM and the analysis mechanism related to inflammation through cellular and molecular experiments.

In addition, this paper has some limitations: 1) the sample size of the research object is small; 2) the inclusion criteria and exclusion criteria of the research objects are not set strictly enough, and they are not well-considered, so it takes a long time to pay a return visit to the research objects in the later stage; and 3) this article does not conduct the research report in full accordance with the STROBE Statement.

$$\text{ICC}=\frac{{\text{Sp}}^{2}+{\text{Sj}}^{2}+{\text{Ss}}^{2}}{{\text{Sp}}^{2}+{\text{Sj}}^{2}+{\text{Ss}}^{2}+{\text{So}}^{2}+{\text{Se}}^{2}}.$$

## Data Availability

The raw data supporting the conclusion of this article will be made available by the authors, without undue reservation.
